# Atlanta Medical Society

**Published:** 1895-03

**Authors:** W. L. Champion

**Affiliations:** Secretary; Atlanta, Ga.


### *SOCIETY REPORTS.*

---

ATLANTA, GA., February 19, 1895.

*The President, Dr. C. D. Hurt, in the Chair.*

DR. J. W. DUNCAN read a paper on the Coal-Tar Derivatives. (See page 17.)

DISCUSSION.

DR. L. H. JONES stated that he had had a reasonable amount of experience with the Coal-tar Derivatives, but the ones he used mostly were phenacetin, antipyrin, and antifebrin. Phenacetin was a reliable antipyretic, but not as good as antipyrin for that purpose. For light pains it was superior to opium, etc., and always obtained desired results and no unpleasant effects. Always used it for neuralgia in preference to opium, although used the opium in cases of deep-seated pain. In cases of fever where they were used to reduce heat they were very reliable, but if used in large doses would produce a profuse perspiration and likely to cause collapse of patient, but had never seen this effect when used in small doses. However, had seen large doses used without any bad effect. Once gave a man antipyrin for neuralgia. Gave him sixty grains; ten grains to a dose to be taken every hour. The man left his office in the morning at ten o'clock and returned about three o'clock in the afternoon. He stated that he took one dose when he obtained the medicine and, as he got all right, he took the balance of it so as to make sure. The man did not suffer any bad effects. Dr. Jones stated that he himself had often taken ten-grain doses for headache without having any bad effects, but did not prescribe this amount. In relieving pain the phenacetin was superior to the antipyrin, and in cases of fever had seen antipyrin fail where the phenacetin had good results. Has used them with good results in cases of rheumatism and had never seen any serious unpleasant effects from any of them, but in one case where antipyrin was used it caused a slight eruption. In cases of children had used all three, and the phenacetin had especially good results in relieving pain, but the antipyrin was the most soluble and for that reason favored prescribing it. It has a peculiar taste but does not remain long and is not disagreeable, and acts on the sensory nerves more than on the motor nerves. All three increase the tendency to take cold, and for that reason should be taken when the person can stay indoors. Had used salol some, but without especially good results.

DR. F. B. MCRAE stated that he was fully convinced of the good of the coal-tar derivatives; that he gave them in place of opiates and almost invariably obtained the desired results. He was not attached more to one than to another; not much difference in them; simply a matter of adaptability of each one to each case, and all very satisfactory. Considered them superior to opiates and therefore used them instead.

DR. J. B. BAIRD was opposed to antikamnia, and never prescribed it, as he understood it to be a composition of drugs. The ones he usually employed were antipyrin and phenacetin, and also antifebrin, but that he preferred the phenacetin to either of the others, as it accomplished the desired results without any unpleasant effects. That they were excellent to reduce the temperature, but that he thought too much importance was attached to the simple existence of heat, and the removing of it, and not enough attention paid to its cause. In a number of cases it was a very good thing to use these heat reducers, as they rendered the patient comfortable and were beneficial in that respect, but there are many conditions where their use is objectionable, as in many diseases where the temperature runs high they should be used with great caution.

DR. L. B. GRANDY stated that in 1894 there were nearly two hundred new preparations, and most of them were coal-tar derivatives, but that he considered it safe to say that not more than one-tenth possess really valuable properties as curative agents or the properties that are claimed for them. The great majority of these preparations will be found to have no advantage over those which have been long in use. The majority of them are synthetic products of the laboratory, and laboratory experiments and reactions are by no means the same as bedside experience and observation; and the doctor's best chance with his patient will be more in the use of remedies which have acquired a fixed place in therapeutics than in all the inventions of the chemists. Still, there are some which are very useful. The three antipyretics have been already discussed. Ichthyol is useful in skin affections, and almost a specific in erysipelas. A saturated alcoholic solution is a good way to use it. Salol has very valuable properties, particularly in diarrheas of children depending upon fermentative changes in the alimentary tract; one or two grains every three hours. Sulphonal is reliable and safe. It rarely fails to produce sleep, its action being slow but generally sure.

DR. R. R. KIME was opposed to the too free use of the coal-tar derivatives in reducing heat in fevers, as in a majority of instances it does not eliminate the cause of the disease, and in many cases damage and injury is done, as they destroy the known methods of ascertaining the condition of the patient, such as by temperature, pulse, etc.

DR. V. O. HARDON stated that of the coal-tar derivatives he used mostly phenacetin, antipyrin, antifebrin, and sulphonal. Used some of the others, but very little. Has used all of the first three mentioned but, as a result of experience, now uses the phenacetin almost entirely. Does not use much antipyretics for the reason stated by Dr. Kime, that it destroys the guide to find the cause of the trouble, by reducing temperature, etc. For neuralgia use phenacetin combined with powdered camphor to reduce the pain, as its energetic effect is increased. Uses sulphonal almost entirely to produce sleep, and it rarely fails.

DR. J. S. TODD stated that when the coal-tar derivatives were first brought out the old physicians used them extensively, as it was gratifying to them to be able to reduce the temperature so easily, and it was shown that the mortality in typhoid fevers increased thirty-five to forty per cent. Many died whose temperature did not rise above 100. All of these remedies are depressing, and any remedy which, given to a patient in typhoid state, takes away vitality is injurious. Glad that they were discovered, but have done great damage in typhoid cases. They are good substitutes for opium and are invaluable in all states of inflammation. In a number of cases it is best not give them, except in conjunction with some stimulant, such as camphor, etc. Do not prescribe them to children, except antipyrin, on account of want of solubility. Used ichthyol for erysipelas, also for gonorrhea, twenty-five per cent. in water. Sulphonal not at all satisfactory.

W. L. CHAMPION, M.D., Secretary.

